# Incidental Paratracheal Air Cysts on Thoracic CT and Their Association with Chronic Inflammatory Lung Disease

**DOI:** 10.1155/2017/8740635

**Published:** 2017-03-15

**Authors:** Ha Yeon Kim, Kyung Hee Lee, Yeo Ju Kim, Ha Young Lee, Ga Ram Kim, Yong Sun Jeon, Jung Soo Kim, Young Sam Kim, Jun Ho Kim

**Affiliations:** ^1^Department of Radiology, Inha University Hospital, Inha University School of Medicine, Inhang-ro 27, Jung-gu, Incheon, Republic of Korea; ^2^Department of Internal Medicine, Inha University Hospital, Inha University School of Medicine, Inhang-ro 27, Jung-gu, Incheon, Republic of Korea; ^3^Department of Thoracic Surgery, Inha University Hospital, Inha University School of Medicine, Inhang-ro 27, Jung-gu, Incheon, Republic of Korea

## Abstract

*Purpose*. To determine the association between the progression of upper lung fibrosis and paratracheal air cysts (PACs) size.* Materials and Methods*. The thoracic CT images of 4573 patients were reviewed for the prevalence, size, and location of PACs and their communication with trachea. In addition, the presence of upper lung fibrosis, emphysema, and bronchiectasis was evaluated in patients with PACs and compared with a control group without PACs. Upper lung fibrosis was analyzed using a fibrosis score system.* Results*. The prevalence of PACs was 6.8%. Communication with tracheal lumen was demonstrated by 31.5% of patients with PACs. The prevalence of fibrosis, emphysema, and bronchiectasis in patients with PACs were 67.5%, 21.9%, and 28.3%, respectively. The prevalence of fibrosis was significantly different in the two groups by univariable and multivariable analysis (odds ratio = 2.077, *P* < 0.001). 140 patients with fibrosis among PAC group underwent a previous or follow-up CT; the prevalence with increase in PAC sizes was higher in patients with increase in fibrosis score than those without it (66.2% versus 17.3%, *P* < 0.001).* Conclusions*. PACs appear to be highly related to upper lung fibrosis and moderately related to bronchiectasis. In patients with fibrosis, PAC sizes tended to increase with the progression of upper lung fibrosis.

## 1. Introduction

Paratracheal air cysts (PACs) are small air collections and are usually detected incidentally by thoracic computed tomography (CT) [1–4]. The incidence of PACs in the general population has been reported to be from 0.75% to 8.1% [3, 5–7].

Most PACs are asymptomatic, but rarely, they are the cause of recurrent infections, chronic cough, right side recurrent laryngeal nerve paralysis, and difficult intubation [3, 4, 7]. PACs are usually located on the right side of tracheal area at the level of the thoracic inlet [2, 4, 6, 8]. Histological findings show that PACs are lined with ciliated columnar epithelia and often communicate with tracheal lumen [5, 6]. PACs and tracheal diverticula are considered similar entities [5]. Tracheal diverticula develop by mucosal herniation through weak points in the trachea due to increased intrathoracic pressure [1, 4, 5].

Some authors have suggested that obstructive lung diseases, such as chronic obstructive pulmonary disease (COPD) and bronchiectasis, are associated with the presence of PACs, but results are debated [1, 2, 6, 7]. In a recent study, it was proposed that upper lung fibrosis could cause traction of the tracheal wall and result in cyst formation [4]. We also considered that upper lung fibrosis might be associated with both development and morphologic changes of PACs. In addition, we previously noticed that pulmonary fibrosis usually accompanies obstructive lung disease in clinical practice [4]. However, little information is available regarding this relationship, and it has not been determined which disease is more associated with the presence of PACs. In fact, no published study has addressed the relation between changes in PAC morphology and degree of upper lung fibrosis [4].

The primary purpose of this study was to determine the incidence and CT features of PACs. The secondary purpose was to explore the relation between PACs and underlying pulmonary disease including upper lung fibrosis, bronchiectasis, and emphysema and to evaluate the association between progression of upper lung fibrosis and PACs size.

## 2. Materials and Methods

This retrospective study was approved by the hospital ethics committee on human studies at our institution.

### 2.1. Patients and CT Imaging Technique

Between January 2014 and June 2014, routine thoracic CT was performed on 4573 patients for different reasons, such as health check-up, pulmonary disease, trauma, lung cancer follow-up, or detection of metastasis.

CT scans were performed using a 16-slice MDCT scanner (Siemens SOMATOM Sensation 16) or a 64-slice MDCT scanner (GE LightSpeed VCT). Thoracic CT scanning was performed from the lower part of the neck to adrenal glands. Axial section data were reconstructed at a thickness of 3 mm using a 3 mm slice interval for the SOMATOM Sensation 16 or at a thickness of 2.5 mm using a 2.5 mm slice interval for the LightSpeed VCT, respectively. All images were processed with standard mediastinal (width, 350 HU; level 20 HU) and lung (width, 1500 HU; level −700 HU) window settings.

### 2.2. Image Analysis

Two radiologists with 8 years (reader 1) and 3 years (reader 2) of experience dedicated to thoracic CT imaging served as independent readers. In cases of disagreement between the two reviewers, consensus was reached by discussion.

PACs were defined as air-attenuations in paratracheal soft tissue with or without communication with tracheal lumen and without communication with lung parenchyma or esophagus. PACs were evaluated based on location (right, left, and bilateral), number (single or multiple), presence of communication with tracheal lumen, and longest diameter on axial CT scans with a lung setting window. In cases with multiple cysts, the largest cyst was included in the statistical analysis.

In all patients with PACs, the presence of fibrosis, emphysema, and bronchiectasis was evaluated. Fibrosis was evaluated in both upper lungs. Upper lung range was defined from apex to carina. To perform detailed analysis, we use the fibrosis scoring system with minimal changes. Four features were included; (1) ground glass opacities, (2) irregular pleural margin, (3) reticular opacities and fibrosis, and (4) honeycombing.

Ground glass opacity was defined as a hazy area of increased attenuation in the lung with preserved bronchial and vascular markings. Irregular pleural margin was defined as pleural thickening with prominent subpleural consolidation opacities. Reticular opacities were defined as a collection of innumerable areas of small linear opacity. Honeycombing was defined as the presence of cystic airspaces measuring 3–10 mm in diameter with 1–3 mm thick walls [9, 10].

Extent of these feature was measured using scoring systems in each upper lung; 0, none; 1, 1–10%; 2, 11–25%; 3, 26–50%; 4, 51–75%; and 5, 76–100% [10]. Scores were summed to calculate fibrosis scores (range 0–20) for each upper lung. Total fibrosis scores were calculated by summing the fibrosis scores of both upper lungs. Using this method minimum and maximum possible total fibrosis scores were 0 and 40, respectively.

The presences of emphysema and bronchiectasis in both lungs (ranging from apices to bases of lower lobes) were evaluated. Emphysema was defined as areas of decreased attenuation with no walls or discrete walls. Bronchiectasis was diagnosed based on bronchial dilatation relative to an adjacent pulmonary artery, lack of bronchial tapering, and visualization of bronchi in lung periphery.

Patients with PACs were classified according to the availability of a previous or follow-up CT. We selected two CT scans with the longest interscan interval. In each of the two scans chosen, the diameter of the largest PAC was measured and upper lung fibrosis score was determined.

We selected 311 patients, matched for age and gender using computer software, as a control group with no PAC. The presences of fibrosis, emphysema, and bronchiectasis in controls were determined as described above.

### 2.3. Statistical Analysis

Student's *t*-test was used to analyze continuous variables and Fisher's exact test or the chi-squared test was used to identify correlations among categorical variables. The Cochran-Armitage test was used to assess linear trends regarding PAC prevalence according to decade of life. Multivariable logistic regression analyses were performed to determine and obtain odds ratios (ORs) of factors affecting the presence and increases in the sizes of PACs. Correlation with fibrosis progression scores and PAC size changes were determined using Spearman's correlation coefficients (rho). The analysis was conducted using SPSS statistical software package (version 19.0, Chicago, IL) and dBSTAT for Windows (version 5.0, Seoul, Korea).* P* values of < 0.05 were considered statistically significant.

## 3. Results

PACs were detected in 311 of the 4573 (6.8%) study subjects, that is, in 184 of 2735 males (6.72%) and in 127 of 1838 females (6.9%), which showed that the prevalence of PACs was not associated with gender (*P* = 0.81). Mean age of the 311 patients with PACs was 63.44 years (±13.55, range 18–107). A list of PAC prevalence against age in decades is presented in Table 1. A plot shows its prevalence increased significantly with age (*P* < 0.001).

Three hundred and eleven patients without PACs were selected as a control group, 184 men and 127 women. Mean age of the control group was 63.45 years (±14.39, range 22–96).

Mean greatest PAC diameter was 5.93 ± 0.21 mm (median 5.00, range 1–22). Most were located in the right lateral side of the trachea (307 of 311, 98.7%); 3 cases were located in the left side (1.9%), and in 1 case of PACs was bilateral. 42 of 311 (13.5%) patients had more than one PAC and 1 patient had multiple PACs located bilaterally with respect to the trachea. 98 of 311 (31.5%) PACs communicated with tracheal lumen (Table 2).

Of the 311 patients with PACs, 210 had upper lung fibrosis (67.5%), 61 had emphysema (21.9%), and 88 had bronchiectasis (28.3%). In the control group, 125 patients had upper lung fibrosis (40.2%), 54 had emphysema (17.4%), and 53 had bronchiectasis (17.0%). Intergroup differences were significant for upper lung fibrosis (*P* < 0.001) and bronchiectasis (*P* < 0.001). The prevalence of fibrosis in patients with PACs was 2.921 (odds ratio, 2.921; 95% CI, 2.077–4.106) times higher than in controls (Table 3).

Of the 311 patients with PACs, 129 of the 184 men (70.1%) and 81 of the 127 women (63.8%) had upper lung fibrosis. No significant differences were observed between genders with respect to the prevalence of upper lung fibrosis (*P* = 0.268). 69 of 98 (70.4%) patient with PACs and tracheal communication had upper lung fibrosis, and 141 of the 213 (66.2%) without communication had upper lung fibrosis, which was not significantly different (*P* = 0.46).

The fibrosis scores of patients with PACs ranged from 1 to 22 and their mean score was 3.53 ± 3.10. In the control group, fibrosis scores ranged from 1 to 15 and the mean score was 3.35 ± 2.34. No significant difference was observed between the fibrosis scores of patients with or without PACs (*P* = 0.50).

**Figure 1:**
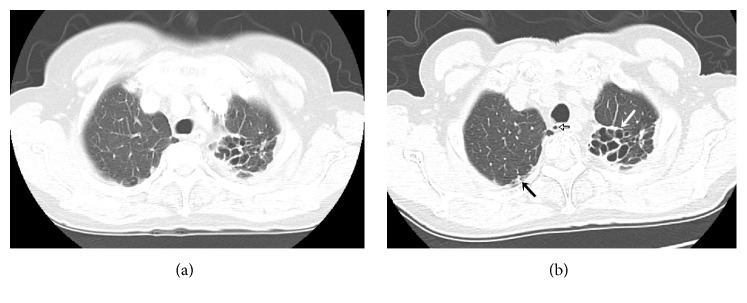
(a) Axial CT scan shows fibrosis and irregular pleural thickening in a left upper lobe. No paratracheal air cyst is visualized. (b) CT scan obtained 57 months later shows a paratracheal air cyst at the right posterior side of the trachea (black line arrow). CT image shows subpleural irregularity in the right upper lobe (black arrow) and progression of fibrosis and bronchiectasis in the left upper lobe (white arrow). The total fibrosis score of both upper lungs increases from 5 to 7 points over the 57 months.

**Figure 2:**
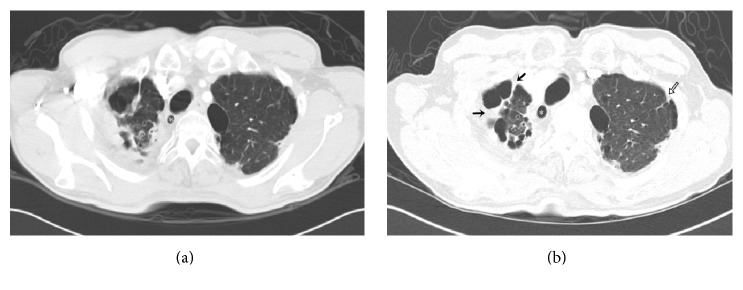
(a) Axial CT scan shows a paratracheal air cyst (*∗*) and fibrosis with volume decrease in a right upper lung and subpleural fibrosis in a left upper lung. (b) CT scan obtained 32 months later shows an increase in PAC size. In addition, fibrosis had thickened (arrow) and the volume of the right upper lobe has reduced. Subpleural fibrosis is more prominent in the left upper lung (black line arrow). The total fibrosis score of both upper lungs increased from 9 to 12 points over the 32 months.

Among 210 patients with upper lung fibrosis, 140 had follow-up CT scans. The mean intervening period was 42.67 months. During this time, upper lung fibrosis scores increased in 56 patients. The mean score change in theses group was 2.57 ± 2.16 points (range 1–13). Of these 56 patients, 43 (76.8%) showed an increase in longest PAC diameter, and the mean diameter increase was 1.73 ± 1.85 mm (range 1–9). In 84 patients without a change in upper lung fibrosis score, 22 (26.2%) showed an increase in longest PAC diameter. 43 of 65 (66.2%) with increased PAC diameter showed progression of upper lung fibrosis (Figure 1), and 17.3% (13 of 75) of those that did not show an increase in diameter showed progression of upper lung fibrosis, and this difference was significant (*P* < 0.001). After adjusting for age, sex, and time to follow-up CT scan, progression of upper lung fibrosis was found to be significantly associated with an increase in PAC size (*P* < 0.001; odds ratio, 8.785; 95% CI, 3.897–19.084) (Table 4). Of the 311 patients with PACs, 15 patients did not have PACs by previous CT. Thirteen of these 15 patients (86.7%) had upper lung fibrosis, and 11 of those 13 patients (84.6%) showed progression of fibrosis (Figure 2).

The correlation between progression of fibrosis score and PAC size change was not significant by Spearman's correlation analysis (*P* = 0.216).

## 4. Discussion

PACs are almost detected incidentally on thoracic CT scans and in most cases are asymptomatic [2]. Previous studies have reported that the prevalence of PACs on CT scan ranges from 2.0% to 8.1% [1, 3–8]. In the present study, which involved the largest numbers of patients with PACs, the prevalence of PACs was 6.8%, which was not surprising, as PACs are not an uncommon finding on thoracic CT scans. This prevalence is higher than that reported by Unlu et al. (5.4%) [4], who used a thicker CT slice, but lower than those reported by Boyaci et al. (8.0%) [7] and Bae et al. (8.1%) [6], who both used a high resolution multidetector CT unit with a slice thickness of 1 mm. Accordingly, it appears that prevalence of PACs as determined by thoracic CT may depend on slice thickness, which suggests that as CT technology evolves reported PAC prevalence will increase.

The etiology of PACs is still the subject of debate; Goo et al. suggested that PACs might be caused by the protrusion of tracheal mucosa through weak points in the trachea due to chronic inflammation or increased intraluminal pressure of trachea [5]. Previous reports have revealed that most PACs are located on the right side of trachea [1, 2, 4–8, 11]. Our findings concur as they were detected on the right side in 307 of the 311 (98.7%) patients. Because the esophagus and aortic arch are usually located on the left side of the trachea, the right side is relatively weaker in terms of withstanding intratracheal pressure, which probably explains reported findings [1, 2, 7].

In the present study, communication with the trachea was observed in 98 patients (31.5%), which is a lower rate than those reported previously (42.6% to 56.1%) [2, 4, 7]. The higher rates observed in these previous studies were probably due to the use of thin CT slices [2, 7] or multiplane analysis [4].

According to our findings, PACs showed a slight but nonsignificant female predominance, and the majority of previous studies have also indicated PACs are more prevalent in women [2, 3, 6–8].

In the present study, mean age of 311 patients with PACs was 63.44 years and the prevalence of PAC was found to peak in the 7th decade of life. Furthermore, we found a positive correlation between age and the presence of PACs (*P* < 0.001). Several authors have examined the relation between the prevalence of PACs and age [1, 6–8]. Boyaci et al. reported a negative correlation between the two [7], but only a small number of subjects were included in this study. In addition, all of the studies conducted, including that by Boyaci et al., reported the prevalence of PACs peaked in the 6th decade [1, 6–8]. These findings suggest that PACs probably do not have a congenital etiology. In a recent report, the prevalence of PACs in pediatric patients (1.3%) was lower than in adults [12], which also suggested the underlying mechanism probably involves an acquired etiology.

Several authors have suggested bronchial diverticula are related to COPD and smoking-related lung diseases [13–16]. Others have suggested that the development of PACs, which is similar to subcarinal or bronchial diverticula, is associated with chronic inflammation and obstructive lung disease (including COPD), upper lung fibrosis, and bronchiectasis [4, 7, 17, 18]. However, these suggested associations remain controversial. Goo et al. [5] and Polat et al. [1] reported that the presence of PACs indicates the presence of obstructive lung disease and possibly emphysema. In addition, Boyaci et al. suggested that the presence of PACs was a significant association with bronchiectasis [7]. In a recent study, it was reported that the prevalence of PACs was associated with upper lobe pulmonary fibrosis [4]. Unlu et al. found the incidence of PACs was significantly associated with upper lobe fibrosis and suggested upper lobe fibrosis contributes to the formation of PACs and that the coexistence of emphysema and fibrosis increases the possibility of the presence of PACs [4]. They also found bronchiectasis was related to the presence of PACs [4]. However, other studies found no relation between emphysematous pulmonary change and the presence of PACs [2, 3, 6–8]. Previous studies have reported controversial results about relationships between PACs and pulmonary diseases. In our study, the presence of PACs was found to be significantly associated with upper lung fibrosis and bronchiectasis, but not with emphysema. Of these two diseases found to be significantly associated with the presence of PACs, only upper lung fibrosis was found to be associated by univariable and multivariable analyses. Bronchiectasis showed a borderline association with PACs (*P* = 0.059) by multivariable analysis. In the present study, upper lung fibrosis was found to be correlated more strongly with the presence of PACs than bronchiectasis or emphysema.

In our study, fibrosis rates of upper lungs are 67.5% in patients with PACs and 40.2% in control group, and this result is higher than rates previously reported by Unlu et al. (45.8%, 19.5%, resp.) [4]. There are several possible reasons for this. First, South Korea is still tuberculosis endemic country and seven times higher than the average incidence of Organisation for Economic Cooperation and Development member countries [19]. The sequelae of tuberculosis represent fibrotic response such as parenchymal bands, apical pleural thickening, or volume loss of the upper lobes [20]. Second, since fibrosis grading system can be applied, it was possible to include our study subjects with delicate fibrosis, compared with previous study [4]. Finally, the use of thin slice CT could affect the rate of upper lung fibrosis as the prevalence of reported PAC. Therefore, the rate of upper lung fibrosis will increase by using CT with thinner slice thickness such as 1 mm or 0.625 mm more than that of our study in both control and PACs groups.

Unlu et al. suggested a significant correlation exists between communication and the presence of upper lobe fibrosis in patients with PACs [4], and in the present study this association was higher for patients with PACs and fibrosis, although no significant difference was found between these patients and nonfibrotic group (70.4% versus 66.2%, resp.).

In some previous studies, grading systems were used to analyze relationships between the presence or morphologic features of PACs and the severities of emphysema or bronchiectasis [6, 7]. In another study, focus was placed on the association between the presence of fibrosis and PACs [4]. In the present study, we modified a previously described semiquantitative fibrosis scoring system to evaluate the presence and progression of lung fibrosis [21] and to determine whether increases in PAC sizes and the progression of upper lobe fibrosis are relevant. Although the mean fibrosis score of patients with PACs was higher than those without PACs, no significant difference was found (3.53 ± 3.10 versus 3.35 ± 2.34, resp.). However, we did find a significant relationship between the progression of fibrosis and an increase in PAC size by both univariable and multivariable analysis. The incidence rate in patients that exhibited upper lung fibrosis progression was 2.921 times higher in those that showed an increase in PAC size than in those that did not. Our results tend to support PACs have an acquired etiology, that is, probably mucosal herniation due to difficulties in expiration associated with chronic inflammation, and indicate upper lung fibrosis contributes to the development of PACs. In the present study, there were 15 newly developed PACs during intervening period. High rates of the presence (86.7%) and progression of upper lung fibrosis (84.6%) with newly developed PACs also suggested that fibrosis play an important role in the occurrence of PACs.

This study had several limitations. First, it is limited by its retrospective nature. In particular, histopathological and bronchoscopic results were not obtained because PACs are an incidental CT finding. Second, diagnoses of upper lung fibrosis, bronchiectasis, and emphysema were based on CT alone and were not confirmed by histopathologic results and pulmonary function tests. Third, because our study group included patients with variable symptoms that underwent thoracic CT, it might be argued that our results better represent the general population. On the other hand, our study subjects better reflected daily practice.

## 5. Conclusions

PACs were found to be positively correlated with age and to show a slight female preponderance. The presence of PACs and upper lung fibrosis were observed to be highly related, and we also observed a borderline association between the presence of PACs and bronchiectasis. In addition, the increase of PAC sizes and the progression of upper lung fibrosis were a significant correlation.

## Figures and Tables

**Table 1:** The prevalence of PACs by decades of life.

Decade of life	Number of subjects	Number of PACs	Prevalence (%)
0~19	82	2	2.4
20~29	113	3	2.65
30~39	265	5	1.88
40~49	654	35	5.35
50~59	1081	74	6.84
60~69	1034	94	9.09
70~79	828	53	6.4
80~89	452	41	9.07
90~109	64	4	6.25
Total	4573	311	6.8

PACs = paratracheal air cysts.

**Table 2:** Cyst characteristics.

PACs (*n* = 311)	
Right paratracheal	307 (98.7%)
Left paratracheal	3 (1.0%)
Both paratracheal	1 (0.3)
Multiple	42 (13.5%)
Communication	98 (31.5%)
Size (mm), mean ± SD	5.93 ± 0.21

*Note*. Data are presented as numbers (percentages) of patients.

SD = standard deviation.

**Table 3:** Demographics and the prevalence of upper lung fibrosis, emphysema, and bronchiectasis in patients with or without PACs.

Variables	PAC+^a^ (*n* = 311)	PACs–^b^ (*n* = 311)	Univariable *P*	Multivariable *P*	Odds ratio (95% CI)
Age (mean ± SD)	63.44 ± 13.55	63.45 ± 14.39	0.989	0.619	
Sex			1	0.795	
Male	184 (59.0%)	184 (59.0%)			
Female	127 (41.0%)	127 (41.0%)			
Upper lung fibrosis	210 (67.5%)	125 (40.2%)	<0.001	<0.001	2.921 (2.07–4.106)
Emphysema	61 (21.9%)	54 (17.4%)	0.157	0.877	
Bronchiectasis	88 (28.3%)	53 (17.0%)	0.001	0.059	

*Note*. All data except *P *values are presented as numbers (percentages) of patients.

PAC = paratracheal air cysts, CI = confidence interval, SD = standard deviation, and *n* = number.

^a^Patients with PACs; ^b^patients without PACs.

**Table 4:** Factors associated with an increase in PAC size.

Variables	Not increase in size of PACs (*n* = 75)	Increase in size of PACs (*n* = 65)	Univariable *P*	Multivariable *P*	Odds ratio (95% CI)
Age (mean ± SD)	64.63 ± 11.93	66.22 ± 13.45	0.460	0.901	
Sex					
Male	49 (65.3%)	40 (61.5%)	0.642	0.829	
Female	26 (34.7%)	25 (38.5%)			
Progression of upper lung fibrosis	13 (17.3%)	43 (66.2%)	<0.001	<0.001	8.785 (3.897–19.084)
Follow-up interval (month)	32 (15–46)	42 (24.5–71)	0.018	0.512	

*Note*. All data except *P *values are presented as numbers (percentages) of patients.

PAC = paratracheal air cysts, CI = confidence interval, SD = standard deviation, and *n* = number.
